# Internet-Based Cognitive Behavioral Therapy for Insomnia (ICBT-i) Improves Comorbid Anxiety and Depression—A Meta-Analysis of Randomized Controlled Trials

**DOI:** 10.1371/journal.pone.0142258

**Published:** 2015-11-18

**Authors:** Yuan-yuan Ye, Yuan-feng Zhang, Jia Chen, Juan Liu, Xun-jun Li, Ya-zhen Liu, Ying Lang, Ling Lin, Xin-Ju Yang, Xiao-Jiang Jiang

**Affiliations:** 1 Department of Neurology, Daping Hospital, Third Military Medical University, Chongqing, China; 2 Department of Neurology, PLA 123 Hospital, Bengbu, PR China; University of Geneva, SWITZERLAND

## Abstract

As the internet has become popularized in recent years, cognitive behavioral therapy for insomnia (CBT-i) has shifted from a face-to-face approach to delivery via the internet (internet-based CBT-i, ICBT-i). Several studies have investigated the effects of ICBT-i on comorbid anxiety and depression; however, the results remain inconclusive. Thus, a meta-analysis was conducted to determine the effects of ICBT-i on anxiety and depression. Electronic databases, including PubMed, EMBASE, PsycINFO and the Cochrane Library (throughout May 28, 2015), were systematically searched for randomized controlled trials (RCTs) of ICBT-i. Data were extracted from the qualified studies and pooled together. The standardized mean difference (SMD) and 95% confidence interval (95% CI) were calculated to assess the effects of ICBT-i on comorbid anxiety and depression. Nine records that included ten studies were ultimately qualified. The effect sizes (ESs) were -0.35 [-0.46, -0.25] for anxiety and -0.36 [-0.47, -0.26] for depression, which were stable using a between-group or within-group comparison and suggest positive effects of ICBT-i on both comorbid disorders. Although positive results were identified in this meta-analysis, additional high-quality studies with larger sample sizes are needed in the future.

## Introduction

Insomnia, which comprises difficulty falling asleep at bedtime or awakening in the middle of the night or too early in the morning, represents a critical public health problem worldwide [1]. In addition to fatigue, insomnia is associated with several detrimental consequences, including mental disorders, low work productivity and a decrease cognitive impairment [2,3]. Anxiety and depression are the most common comorbid mental symptoms of insomnia and also exacerbate the sleep disorder [4]. To treat insomnia, medications (especially sedative hypnotics) have been used because of their quick effects in a short time period [5]. However, medication for insomnia can present several adverse effects, such as headache and dizziness during short-term use [6] and addiction during long-term use [7]. Moreover, medication has a limited effect on the long-term relief of insomnia [8].

Cognitive behavioral therapy (CBT), which has recently been proposed as a first-line approach for the treatment of insomnia, primarily includes cognitive strategies (addressing dysfunctional beliefs regarding sleep and education related to sleep health) and behavioral strategies (stimulus control [SC], sleep restriction [SR], relaxation) [9]. CBT approach for insomnia (CBT-i) could improve poor sleep on several aspects (such as sleep latency, total sleep time, etc.), as well as insomnia-related anxiety and depression [10]. Evidence has indicated that the effects of CBT for insomnia have similar short-term but better long-term outcomes than pharmacological interventions [11]. However, traditional CBT (conducted via a face-to-face approach) has several disadvantages, including the relative lack of therapists, time and geographic limitations, and high costs [12]. With the development of internet technology, an innovative and interactive solution has been developed to deliver CBT via the internet, which removes the disadvantages and inconveniences of the traditional approach. The use of internet-based CBT for insomnia (ICBT-i) enables patients with insomnia to obtain treatment and communicate with the therapist at any time and place. Patients can also review announcements and the therapeutic schedule as desired. ICBT-i also reduces the therapist’s time commitment and increases the treatment efficiency. In a previous randomized controlled trial (RCT) on insomnia, Ström and colleagues provided evidence that ICBT-i significantly improved sleep onset latency (SOL), wake time after sleep onset (WASO) and sleep efficiency (SE) [13]. Moreover, the results of recent systematic reviews and meta-analyses have indicated that ICBT-i promotes a significant improvement in the number of awakenings (NOA), SOL and SE [14]. Despite the confirmed efficacy, the effects of ICBT-i on comorbid anxiety and depression remain inconclusive. Thus, a meta-analysis was conducted to assess the efficacy of ICBT-i on comorbid anxiety and depression by pooling published randomized controlled trials (RCTs).

## Materials and Methods

### Database search

This study was conducted according to the Preferred Reporting Items for Systematic Reviews and Meta-Analyses criteria (PRISMA) [15] as shown in S1 File. Electronic databases, including PubMed, EMBASE, PsycINFO and the Cochrane Library (through May 28, 2015), were systemically searched for potential records. The following combination of key words was used in the search strategy: (internet OR web OR online OR computer OR computerized) AND (CBT OR cognitive behavioral therapy OR cognitive therapy OR behavioral therapy) AND (insomnia OR sleep disorder OR sleep problem) AND (RCT OR randomized controlled trial OR clinical trial). Other relevant studies (related references and conference abstracts) were manually identified. When more than one study contained the same population and data, only the most recent or most complete study was included.

### Inclusion and exclusion criteria

The inclusion criteria for the studies in the present meta-analysis were as follows: 1) studies that investigated the effects of ICBT-i on comorbid anxiety, depression or both symptoms; 2) RCTs; 3) the participants were adults (>18 years) diagnosed with either primary or secondary insomnia or a sleep difficulty that occurred at least 3 nights per week and was present for at least 4 weeks; 4) sufficient data were available to calculate the effect size (ES) and 95% confidence interval (95% CI); and 5) studies were published in full text. For this study, the CBT approach was defined as the incorporation of behavioral strategies (SC and SR) and cognitive strategies (addressing dysfunctional beliefs regarding sleep and education related to sleep health) with treatment in more than one session. The following exclusion criteria were used: 1) systemic reviews; 2) non-RCTs; 3) studies that did not include insomnia-linked anxiety and depression; 4) ICBT-i treatment via a gaming machine, DVDs, telephone, or other methods without communication with therapists; or 5) repeated or overlapping studies.

### Inter-rater agreement

The retrieved records were independently screened by two reviewers. After screening by title and abstract, the full texts of the remaining records were further assessed according to the inclusion criteria. Disagreements were resolved by consensus. If the two reviewers could not reach a consensus, another reviewer was consulted. The inter-rater reliability was examined via the kappa test [16].

### Data extraction

Information was extracted using a predesigned data form. The following terms were collected from each qualified study: first author, publication date, sample size, gender, mean age with standard deviation (SD), type of insomnia, medical comorbidity, therapeutic components, treatment duration, questionnaire or rating scale for anxiety and depression, and mean and SD scores of anxiety and depression in both the ICBT-i and control groups (pre- and post-treatment). If the study only provided the standard error, the corresponding SD was calculated. Three sleep parameters, including the SOL, total sleep time (TST), and SE, were also extracted.

### Quality assessment

The quality of eligible articles was evaluated by Jadad score [17]. Two authors performed the assessment independently. As the two authors could not reach a consensus, the corresponding author was consulted.

### Data analysis

The effects of ICBT-i on anxiety and depression were measured using the ES of Cohen’s d value and the 95% CI. When studies included valid data regarding anxiety and/or depression in the control group, the between-group ES (the difference between the experimental and control groups post-treatment) was computed. Otherwise, the within-group ES (the difference between pre- and post-treatment for the experimental group) was computed. To obtain the ES with the 95% CI, a random effects model was used when heterogeneity was present (I^2^>50%, p<0.05), and a sensitivity analysis was subsequently performed to examine the source of the heterogeneity. Otherwise, a fixed effects model was used [18]. Statistical analyses were performed using STATA statistical software (version 12; Stata Corporation, College Station, Texas).

### Sensitivity analysis and publication bias

Sensitivity analysis was conducted by removing the included studies one by one and estimating the overall effects size. Publication bias was assessed by funnel plot (using Review Manager 5.3) and Egger’s test (using STATA 13.0). If the funnel plot was symmetrical and the P value was >0.05, it indicated no publication bias.

## Results

### Qualified studies and study characteristics

**Fig 1:**
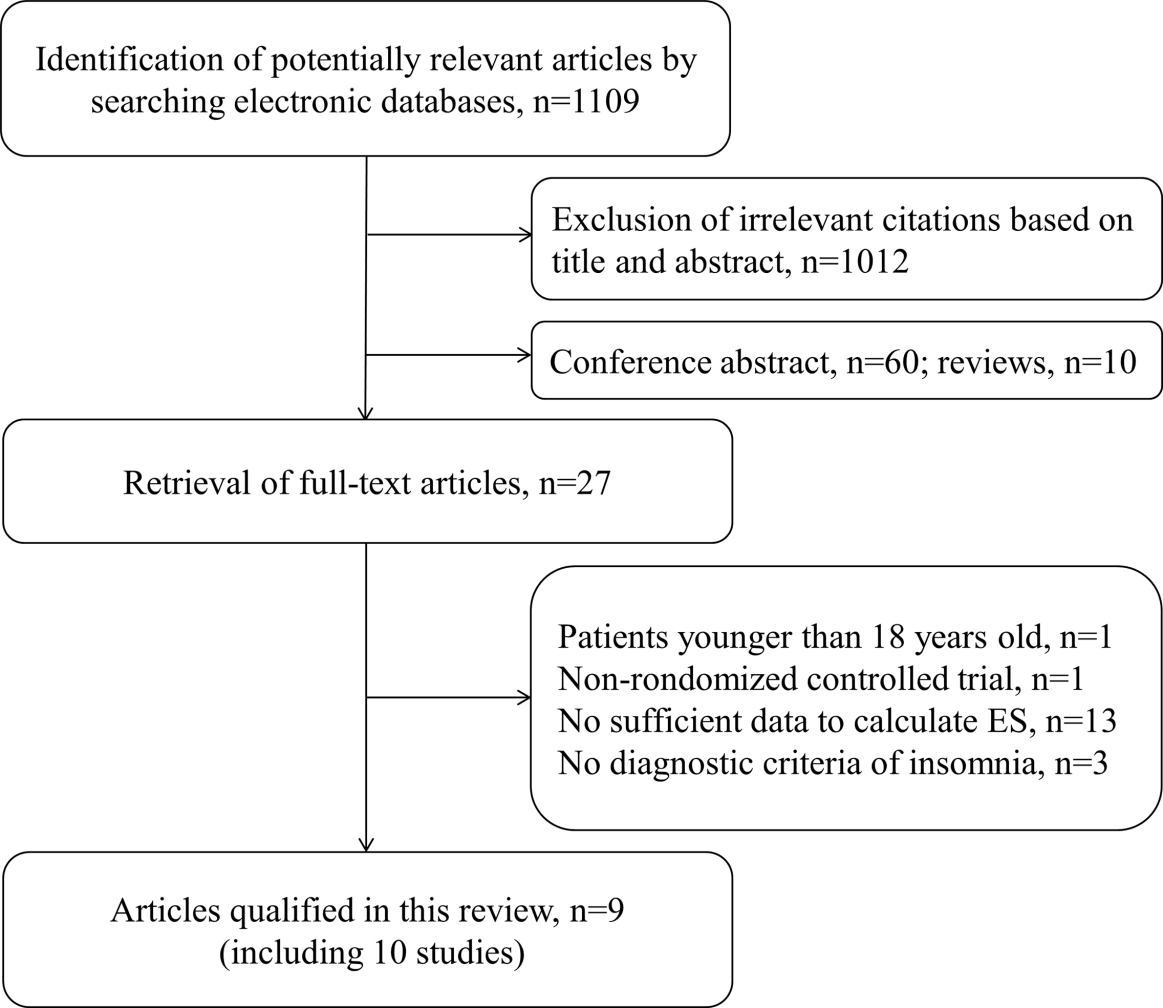
Flow diagram of the qualified studies.

The detailed selection procedure is shown in Fig 1. A total of 1109 records were retrieved from the online databases. After screening, 1012 records were excluded according to the titles and abstracts; 60 records contained conference abstracts, and 10 records comprised reviews, which resulted in 27 records for full-text review. Of these full-text records, one record [19] was excluded because the participants were <18 years of age, one record [20] was a non-RCT, and three records [21–23] did not provide the diagnostic criteria for insomnia or lacked sufficient information regarding the diagnosis of insomnia. Moreover, thirteen other records did not report sufficient data concerning anxiety and/or depression. Ultimately, 9 records [24–32] that comprised 10 studies (eight studies regarding anxiety and depression [24–30] and two studies regarding only depression [31–32]) were included in this meta-analysis. There was perfect inter-rater reliability during the selection process, with kappa values of 0.84 based on the titles, 0.88 based on the abstracts and 0.92 based on the full text.

The main characteristics of the included studies are shown in Table 1. Most studies comprised mixed subjects with primary or secondary sleep disorders; however, one study [27] comprised subjects with only a primary sleep disorder. Different components (such as sleep hygiene education [SHE], cognitive restructuring [CR], SC, and SR) were applied in the ICBT-i procedure for each study. Three studies [25,27,31] had a nine-week duration of therapy, one study [32] had an eight-week duration of therapy, and the other six studies had a six-week duration of therapy. Anxiety and/or depression were measured using four main rating scales, including the Hospital Anxiety and Depression Scale (HADS) [33,34], the Centre of Epidemiological Studies Depression Scale (CES-D) [35,36], the State-Trait Personality Inventory (STPI) [37], the Depression Anxiety and Stress Scales (DASS)[38] and the Montgomery Asberg Depression Rating Scale (MADRS-S) [39]. Table 2 shows the sample characteristics and the data regarding anxiety and depression extracted from the eight studies. Seven hundred seventy-six experimental cases (female: 69.2%) and 454 controls in the waiting list (female: 72.0%) were included. The rate of dropout was calculated for each study, with an average of 19.3% for the experimental group and 15.6% for the waiting list.

**Table 1:** Study characteristics.

Study	Sleep disorder	ICBT-i components	Duration	Rating scale (reference)
Lancee, 2012 [22]	P. INS & S. INS	SHE-CR-SC-SR-PI	6w	HADS(Spinhoven et al.1997); CES-D(Bouma et al.1995)
Ritterband, 2012 [23]	P. INS & S. INS	SHE-CR-SC-SR	9w	HADS (Zigmond et al.1983)
Lancee, 2013a [24]	P. INS & S. INS	SHE-CR-SC-SR-RLX-PI	6w	HADS(Spinhoven et al.1997); CES-D(Bouma et al.1995)
Lancee, 2013b [24]	P. INS & S. INS	SHE-CR-SC-SR-RLX-PI	6w	HADS(Spinhoven et al.1997); CES-D(Bouma et al.1995)
Thorndike, 2013 [25]	P. INS	SHE-CR-SC-SR	9w	STPI(Spielberger et al.1995)
Espie, 2014 [26]	P. INS & S. INS	SHE-CR-SC-SR-RLX-PI	6w	DASS(Henry et al.2005)
Ho, 2014 [27]	P. INS & S. INS	SHE-CR-SC-SR-RLX	6w	HADS(Zigmond et al.1983)
van Straten, 2014 [28]	P. INS & S. INS	SHE-CR-SC-SR-RLX	6w	HADS(Spinhoven et al.1997); CES-D(Beekman et al.1997)
Blom, 2015a [29]	P. INS & S. INS	SHE-CR-SC-SR- RLX	9w	MADRS-S(Montgomery et al.1979)
Blom, 2015b [30]	P. INS & S. INS	SHE-CR-SC-SR- RLX	8w	MADRS-S(Montgomery et al.1979)

Note: P. INS = Primary insomnia; S. INS = Secondary insomnia; SHE = Sleep hygiene education; CR = Cognitive restructuring; SC = Stimulus control; SR = Sleep restriction; PI = Paradoxical intention; RLX = Relaxation; HADS = Hospital Anxiety and Depression Scale; CES-D = Centre of Epidemiological Studies Depression scale; STPI = State-Trait Personality Inventory; DASS = Depression Anxiety and Stress Scales; MADRS-S = Montgomery Asberg Depression Rating Scale.

**Table 2:** Sample characteristics and rating scores of comorbid anxiety and depression.

Study	Group	N(%Female)	Mean	Anxiety		Depression		%
			age(SD)	Pre-test	Post-test	Pre-test	Post-test	Dropout
Lancee, 2012	Internet	214(68.7)	52.2(11.4)	5.63(3.19)	4.26(2.84)	14.29(6.47)	10.67(7.08)	22.9
	Waiting list	200(68.0)	51.9(12.2)	5.46(3.23)	5.28(3.41)	13.12(6.26)	11.81(7.08)	9.0
Ritterband, 2012	Internet	14(100)	53.7(10.8)	9.43(4.29)	6.71(3.85)	5.21(3.58)	3.21(2.42)	0
	Waiting list	14(71.4)	59.6(12.3)	8.57(3.27)	7.50(2.98)	5.43(2.65)	5.14(4.02)	0
Lancee, 2013a	Internet	133(73.7)	47.38(11.83)	6.99(3.78)	5.98(3.93)	18.39(10.07)	15.20(10.78)	22.0
Lancee, 2013b	Internet+email	129(76.7)	49.33(13.19)	7.08(3.96)	4.82(3.30)	17.29(9.31)	11.31(8.18)	10.9
Thorndike, 2013	Internet	22(81.8)	44.68(10.61)	19.77(4.32)	17.32(3.70)	18.62(4.27)	16.23(5.34)	4.5
	Waiting list	22(72.7)	45.05(11.67)	17.64(4.01)	18.55(4.71)	17.32(4.31)	18.27(4.05)	0
Espie, 2014	Internet	55(72.7)	50.7 (13.8)	2.32(2.00)	1.74(2.30)	4.98(2.97)	3.38(3.11)	21.82
	Waiting list	54(70.4)	49.1 (13.7)	3.29(2.57)	2.98(2.65)	5.53(3.38)	4.53(3.31)	12.96
Ho, 2014	Internet	104(67.3)	38.6(11.8)	9.20(4.08)	8.80(5.10)	6.90(4.08)	6.70(5.09)	42.3
	Waiting list	105(75.2)	39.9(12.7)	9.90(4.10)	9.10(5.12)	7.60(4.10)	7.60(5.12)	38.1
van Straten, 2014	Internet	59(59.3)	48.7(13.8)	4.40(2.60)	3.20(2.80)	12.00(6.60)	8.80(7.10)	16.9
	Waiting list	59(81.4)	50.1(11.9)	4.80(2.20)	4.70(2.90)	12.80(7.00)	11.80(6.40)	10.2
Blom, 2015a	Internet	22 (36.4)	46.1 (13.6)	-	-	25.1 (5.9)	18.7 (11.4)	9.1
Blom, 2015b	Internet	24 (33.3)	56.1 (10.2)	-	-	12.5 (7.5)	7.7 (6.1)	4.2

### Quality assessment

All studies included in our meta-analysis were high quality (Jadad score = 3). Of these, the random method and attrition details were mentioned. All included studies did not describe complete blinding of all participants and personnel, and only one study [29] reported complete blinding of outcome assessment.

### ES summary data

**Fig 2:**
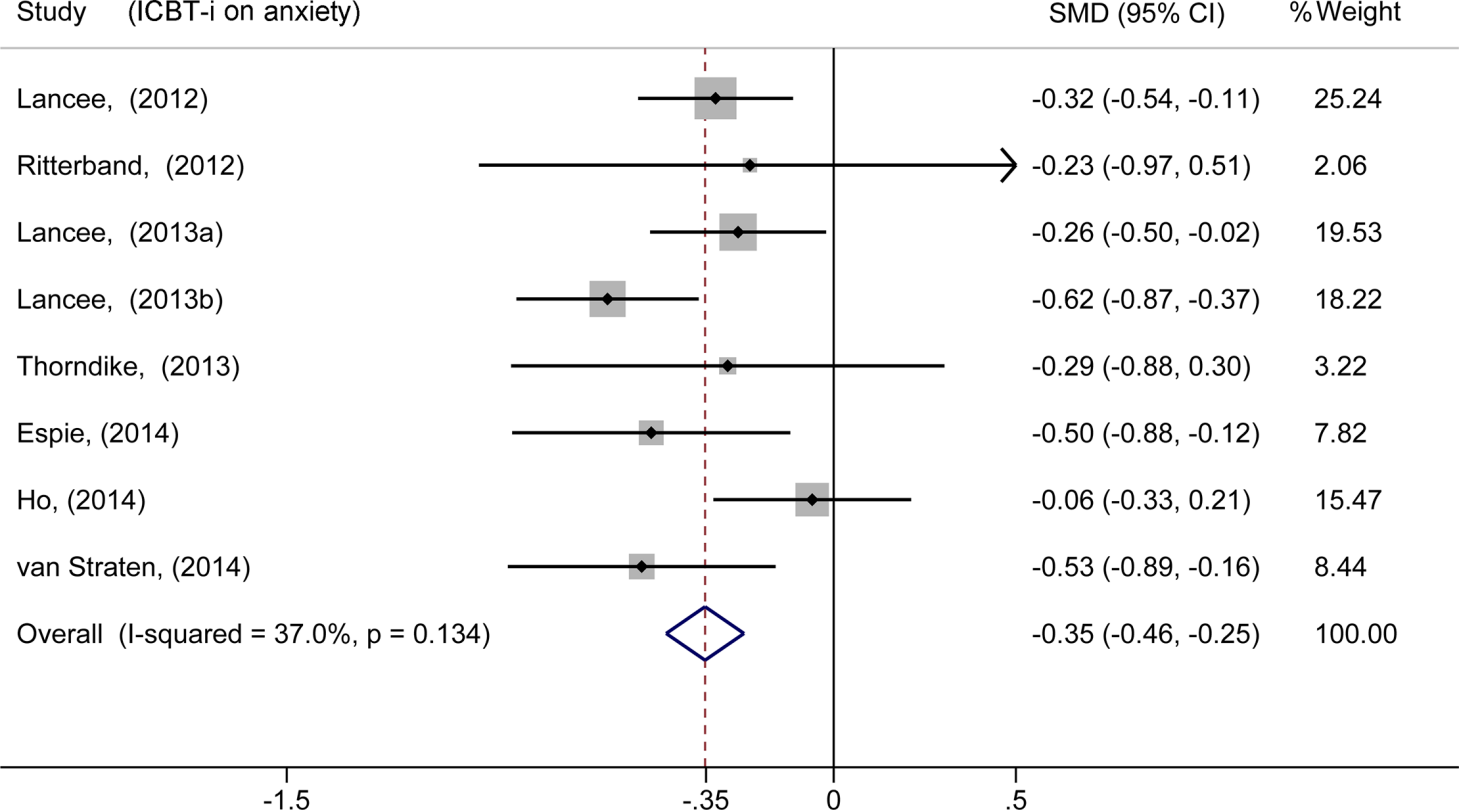
Meta-analysis of the effect of ICBT-i on anxiety.

**Fig 3:**
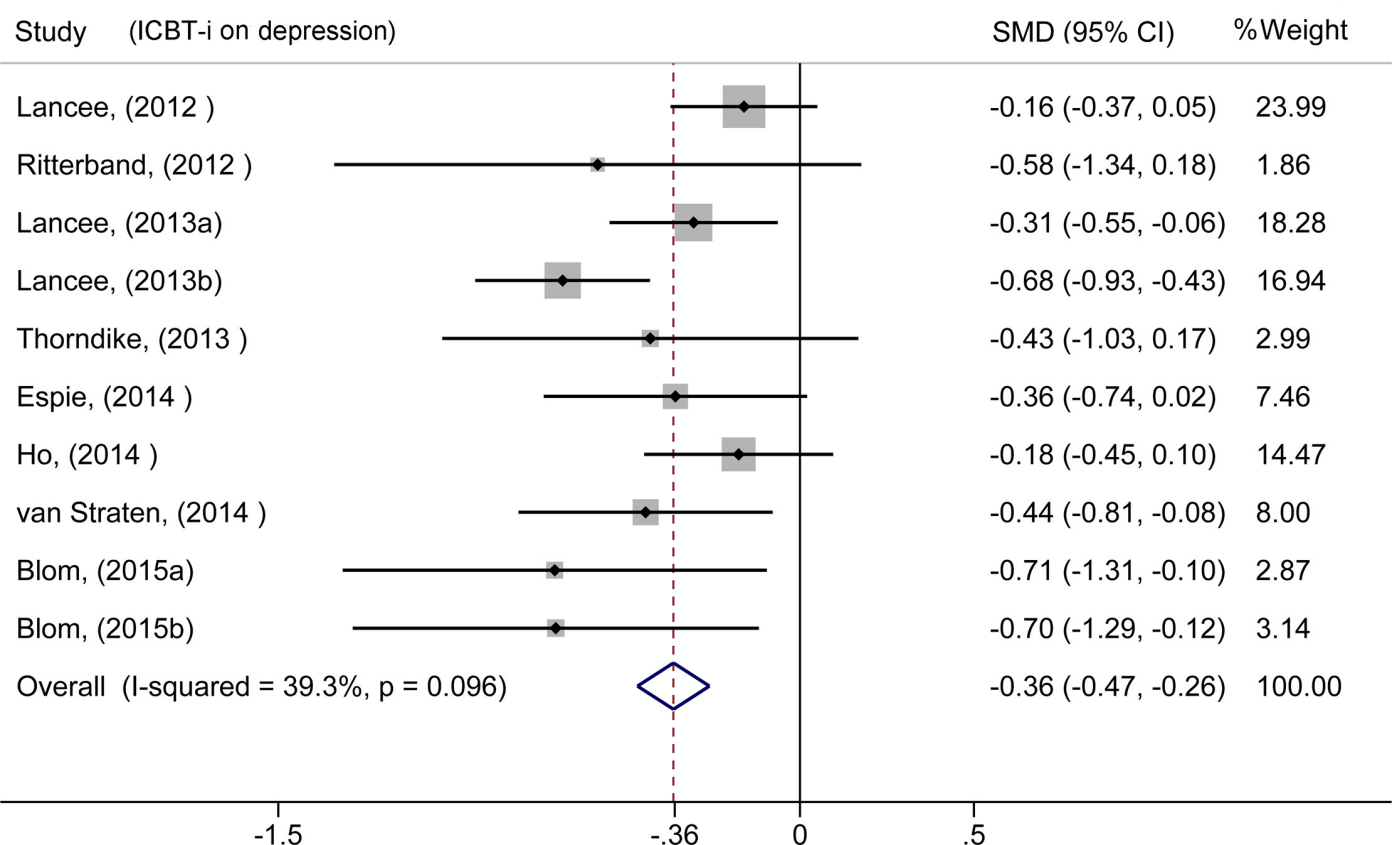
Meta-analysis of the effect of ICBT-i on depression.

We first investigated the effects of ICBT-i, and the combined ESs were -0.50 [-0.72, -0.28] for the SOL, 0.41 [0.19, 0.64] for the TST and 0.75 [0.48, 1.01] for the SE, which indicates positive effects of ICBT-i. The standardized mean difference (SMD) was applied while pooling the rating score data. For anxiety, three studies[25,27,29] had non-significant ESs; however, the other five studies exhibited positive results. Following the meta-analysis, the summary ES was -0.35 [-0.46, -0.25], which suggests a positive effect of ICBT-i on comorbid anxiety (Fig 2). We also calculated the ES of the within-group comparisons (-0.38 [-0.49, -0.28]) and between-group comparisons (-0.30 [-0.44, -0.17]), both of which indicated a positive effect of ICBT-i on comorbid anxiety. For depression, five studies[24,25,27–29] had non-significant ESs; however, the other five studies exhibited positive results. Following the meta-analysis, the summary ES was -0.36 [-0.47, -0.26], which suggests a positive effect of ICBT-i on comorbid depression (Fig 3). The combined ES of the within-group comparisons was -0.45 [-0.55, -0.35], whereas the combined ES of the between-group comparisons was -0.27 [-0.41, -0.13].

To determine the changes in anxiety and depression in the waiting list, the combined ESs were calculated using within-group comparisons for both anxiety (-0.08 [-0.22, 0.05]) and depression (-0.40 [-0.95, 0.16]). The findings indicated there was no improvement in anxiety or depression in the waiting list.

### Sensitivity analysis and publication bias

Sensitivity analysis showed that the results of ICBT-i on comorbid anxiety and depression were relatively stable (S1 Fig). The funnel plots were symmetrical as shown in S2 Fig, Egger’s test quantitatively revealed no significant publication bias (for anxiety, P = 0.925; for depression, P = 0.169).

## Discussion

By pooling the data obtained from qualified RCTs, we identified evidence that ICBT-i was effective in improving comorbid anxiety and depression with a mild strength (summary ES: -0.35 [-0.46, -0.25] for anxiety, -0.36 [-0.47, -0.26] for depression), which was consistent with the results regarding the effect of ICBT-i derived from this study (ES: -0.50 [-0.72, -0.28] for the SOL, 0.41 [0.19, 0.64] for the TST and 0.75 [0.48, 1.01] for the SE).

The internet connects individuals beyond geographical limitations. In contrast to traditional CBT, which has been proposed as the best therapy for insomnia and requires face-to-face interactions with therapists [40], patients with insomnia can receive ICBT-i treatment using a computer and other electronic devices (iPad, iPhone). These devices allow patients to access the interactive website or software, which has friendly interfaces and provides relevant media. In addition, patients with insomnia can communicate with their therapist in real time. The ICBT-i approach also has an increased cost-benefit compared with traditional methods [22]. Most importantly, this remote, self-help approach to therapy can help patients learn how to treat themselves in cases of recurrence. ICBT-i, which has been demonstrated to improve different aspects of insomnia, such as the SOL, TST and SE, is a novel and effective therapeutic modality for insomnia [41]. Based on the data from the RCTs, we investigated the effects of iCBT-i, which were consistent with the previous meta-analysis [13]. Anxiety and depression are the most common mood disorders comorbid with insomnia [42]. Anxiety and depression, in turn, can also exacerbate the symptoms of insomnia [43]. Thus, traditional CBT therapy for insomnia should theoretically improve comorbid anxiety and depression. Several studies had shown efficacious effects of traditional CBT-i on the comorbid mood disorders [10,44,45]. A meta-analysis published in 2011, which included 50 studies, had found that CBT-i had a small to moderate effect on concomitant anxiety (0.406 [0.318, 0.493]) [46]. In addition, several studies had also suggested that traditional CBT-i could improve the comorbid depression [10,45,47]. However, whether ICBT-i is efficacious for comorbid anxiety and depression requires additional evidence. By including RCTs that address this issue, we determined that ICBT-i was effective in improving both comorbid anxiety and depression. This result was in accordance with findings that demonstrate the effect of ICBT-i, which supports the connection between insomnia and mood disorders.

Although the RCTs included in our study were of high quality and the results were relatively stable, there was heterogeneity that may have influenced the final results. First, the sample sizes of several included studies were small. Moreover, ICBT-i therapy varied in the number and duration of therapy sessions, which may have yielded different efficacies in improving insomnia and comorbid symptoms. Furthermore, rating scales with different sensitivities were applied to assess anxiety and depression, which may have resulted in inconsistent outcomes [48]. Other confounding factors include the rates of dropout in the different studies (which ranged from 0 to 42.3% in this meta-analysis) and the initial level of comorbid anxiety and depression at patient recruitment. As an independent factor, time should be considered a critical aspect in the assessment of therapeutic efficacy [49]. However, time had no effect on anxiety or depression according to the within-group comparisons of the waiting list (0.08 [-0.22, 0.05] for anxiety and 0.40 [-0.95, 0.16] for depression in this meta-analysis). Finally, most studies included in this meta-analysis used a control group, and the ES was therefore calculated by a between-group comparison; however, for the two studies without a qualifying control group, the ES was calculated by a within-group comparison. The heterogeneity derived from these potentially confounding factors may have affected the reliability of the final results. For methodological reasons, several limitations existed in this meta-analysis. First, the potential studies searched were published in English. Second, unpublished studies were not included. Moreover, although publish bias was not observed in the present meta-analysis, some unpublished studies might be important.

In summary, we identified an effect of ICBT-i on comorbid anxiety and depression, despite the heterogeneity and limitations of the meta-analysis. Additional high quality studies with larger sample sizes are needed to provide further evidence.

## Supporting Information

S1 Fig Sensitivity analysis of the effects of ICBT-i on anxiety and depression. (TIF)

S2 Fig Funnel plot of publication bias (ICBT-i on anxiety and depression). (TIF)

S1 File PRISMA checklist. (DOC)
