# Supplementary figures and images for: Internet-Based Cognitive Behavioral Therapy for Insomnia (ICBT-i) Improves Comorbid Anxiety and Depression—A Meta-Analysis of Randomized Controlled Trials
Source: PLoS One. 2015 Nov 18;10(11):e0142258. doi: 10.1371/journal.pone.0142258 (PMC4651423; doi:10.1371/journal.pone.0142258)

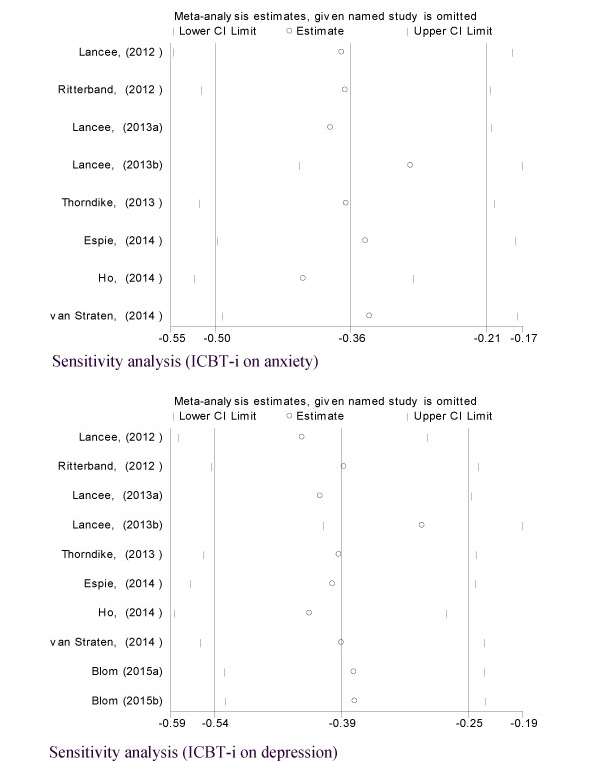

Supplement: S1 Fig — (TIF) [file pone.0142258.s001.tif]

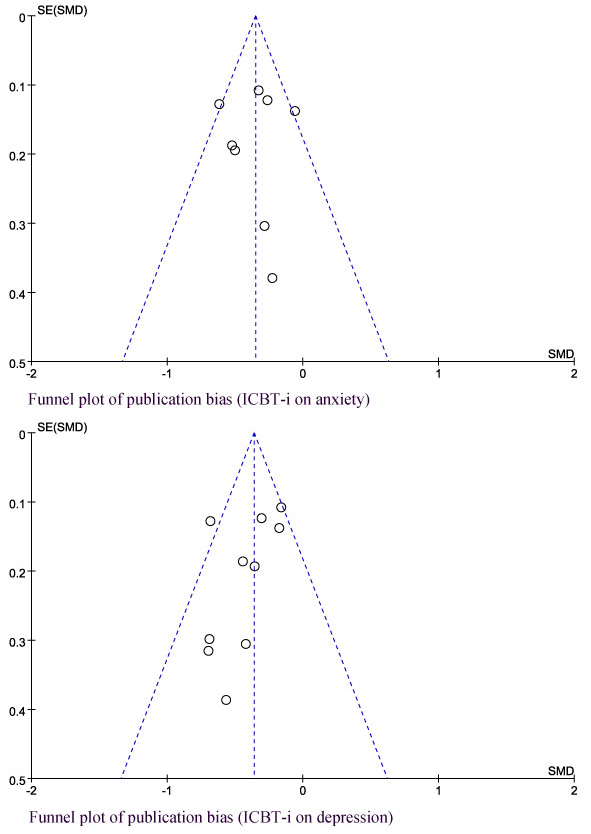

Supplement: S2 Fig — (TIF) [file pone.0142258.s002.tif]
