# Supplementary material for: Internet-Based Cognitive Behavioral Therapy for Insomnia (ICBT-i) Improves Comorbid Anxiety and Depression—A Meta-Analysis of Randomized Controlled Trials
Source: PLoS One. 2015 Nov 18;10(11):e0142258. doi: 10.1371/journal.pone.0142258 (PMC4651423; doi:10.1371/journal.pone.0142258)
Supplement: S1 File — (DOC) [file pone.0142258.s003.doc]

| **Section/topic** | **#** | **Checklist item** | **Page #** |
| --- | --- | --- | --- |
| **TITLE** | | |  |
| Title | 1 | Internet-based cognitive behavioral therapy for insomnia (ICBT-i) improves comorbid anxiety and depression – a meta-analysis of randomized controlled trials | 1 |
| **ABSTRACT** | | |  |
| Structured summary | 2 | **Background:** As the internet has become popularized in recent years, cognitive behavioral therapy for insomnia (CBT-i) has shifted from a face-to-face approach to delivery via the internet (internet-based CBT-i, ICBT-i). Several studies have investigated the effects of ICBT-i on comorbid anxiety and depression; however, the results remain inconclusive. Thus, a meta-analysis was conducted to determine the effects of ICBT-i on anxiety and depression.  **Methodology/Principal Findings:** Electronic databases, including PubMed, EMBASE, PsycINFO and the Cochrane Library (throughout May 28, 2015), were systematically searched for randomized controlled trials (RCTs) of ICBT-i. Data were extracted from the qualified studies and pooled together. The standardized mean difference (SMD) and 95% confidence interval (95% CI) were calculated to assess the effects of ICBT-i on comorbid anxiety and depression. Nine records that included ten studies were ultimately qualified. The effect sizes (ESs) were -0.35 [-0.46, -0.25] for anxiety and -0.36 [-0.47, -0.26] for depression, which were stable using a between-group or within-group comparison and suggest positive effects of ICBT-i on both comorbid disorders.  **Conclusions/Significance:** This meta-analysis identified an effect of ICBT-i on comorbid anxiety and depression, despite the heterogeneity and limitations of the meta-analysis. Additional high quality studies with larger sample sizes are needed to provide further evidence. | 2 |
| **INTRODUCTION** | | |  |
| Rationale | 3 | Cognitive behavioral therapy (CBT), which has recently been proposed as a first-line approach for the treatment of insomnia, primarily includes cognitive strategies (addressing dysfunctional beliefs regarding sleep and education related to sleep health) and behavioral strategies (stimulus control [SC] and sleep restriction [SR]). Evidence has indicated that the effects of CBT for insomnia have similar short-term but better long-term outcomes than pharmacological interventions. However, traditional CBT (conducted via a face-to-face approach) has several disadvantages, including the relative lack of therapists, time and geographic limitations, and high costs. With the development of internet technology, an innovative and interactive solution has been developed to deliver CBT via the internet, which removes the disadvantages and inconveniences of the traditional approach. The use of internet-based CBT for insomnia (ICBT-i) enables patients with insomnia to obtain treatment and communicate with the therapist at any time and place. Patients can also review announcements and the therapeutic schedule as desired. ICBT-i also reduces the therapist’s time commitment and increases the treatment efficiency. | 3 |
| Objectives | 4 | The present meta-analysis was conducted to assess the efficacy of ICBT-i on comorbid anxiety and depression by pooling published randomized controlled trials (RCTs). | 3 |
|  | | |  |
| **METHODS** | | |  |
| Protocol and registration | 5 | No protocol and registration. |  |
| Eligibility criteria | 6 | 1) studies that investigated the effects of ICBT-i on comorbid anxiety, depression or both symptoms; 2) RCTs; 3) the participants were adults (>18 years) diagnosed with either primary or secondary insomnia or a sleep diﬃculty that occurred at least 3 nights per week and was present for at least 4 weeks; 4) sufficient data were available to calculate the effect size (ES) and 95% confidence interval (95% CI); and 5) studies were published in full text. | 4 |
| Information sources | 7 | Electronic databases, including PubMed, EMBASE, PsycINFO and the Cochrane Library (through May 28, 2015), were systemically searched for potential records. | 3 |
| Search | 8 | The following combination of key words was used in the search strategy: (internet OR web OR online OR computer OR computerized) AND (CBT OR cognitive behavioral therapy OR cognitive therapy OR behavioral therapy) AND (insomnia OR sleep disorder OR sleep problem) AND (RCT OR randomized controlled trial OR clinical trial). | 3-4 |
| Study selection | 9 | Two investigators (Ye YY and Liu J) screened each of the titles, abstracts, and full texts to determine inclusion independently. The results were compared and disagreements were resolved by consensus. | 4 |
| Data collection process | 10 | Information was carefully extracted from all included publications independently by two of the authors (Ye YY and Liu J) according to the inclusion criteria listed above. Disagreement was resolved by consensus. If these two authors could not reach a consensus, another author (Jiang XJ) was consulted. | 4 |
| Data items | 11 | The following terms were collected from each qualified study: first author, publication date, sample size, gender, mean age with standard deviation (SD), type of insomnia, medical comorbidity, therapeutic components, treatment duration, questionnaire or rating scale for anxiety and depression, and mean and SD scores of anxiety and depression in both the ICBT-i and control groups (pre- and post-treatment). | 4 |
| Risk of bias in individual studies | 12 | The quality of included studies was evaluated independently by two authors (Ye YY and Liu J). | 4 |
| Summary measures | 13 | The principal summary measures are standardized mean differences (SMDs) and 95% confidence intervals (CIs). | 5 |
| Synthesis of results | 14 | The strength of efficacy of ICBT-i on anxiety and depression was measured by effect size of SMD and 95%CIs. The combined ESs were calculated respectively for anxiety and depression. The ESs were pooled through a fixed effects model when no heterogeneity was observed among studies. Otherwise, a random effects model was adopted. | 5 |

| Risk of bias across studies | 15 | Publication bias was assessed by funnel plot (using Review Manager 5.3) and Egger’s test (using STATA 13.0). | 5 |
| --- | --- | --- | --- |
| Additional analyses | 16 | Analysis of efficacy of ICBT-i on insomnia was conducted. Within-group and between-group comparisons were also performed. | 5 |
| **RESULTS** | | |  |
| Study selection | 17 | A total of 1109 records were retrieved from the online databases. After screening, 1012 records were excluded according to the titles and abstracts; 60 records contained conference abstracts, and 10 records comprised reviews, which resulted in 27 records for full-text review. Ultimately, 9 records that comprised 10 studies (eight studies regarding anxiety and depression and two studies regarding only depression) were included in this meta-analysis. | 6 |
| Study characteristics | 18 | Table 1 shows the studies included in the meta-analysis and their main characteristics. | 13 |
| Risk of bias within studies | 19 | There was perfect inter-rater reliability during the selection process, with kappa values of 0.84 based on the titles, 0.88 based on the abstracts and 0.92 based on the full text. | 6 |
| Results of individual studies | 20 | The main results of individual studies were shown in Figure 2. |  |
| Synthesis of results | 21 | The effect sizes (ESs) were -0.35 [-0.46, -0.25] for anxiety and -0.36 [-0.47, -0.26] for depression, which were stable using a between-group or within-group comparison and suggest positive effects of ICBT-i on both comorbid disorders. | 7 |
| Risk of bias across studies | 22 | The funnel plots were symmetrical as shown in S2 Fig, Egger’s test quantitatively revealed no significant publication bias. | 8 |
| Additional analysis | 23 | We first investigated the effects of ICBT-i, and the combined ESs were -0.50 [-0.72, -0.28] for the SOL, 0.41 [0.19, 0.64] for the TST and 0.75 [0.48, 1.01] for the SE, which indicates positive effects of ICBT-i. To determine the changes in anxiety and depression in the waiting list, the combined ESs were calculated using within-group comparisons for both anxiety (-0.08 [-0.22, 0.05]) and depression (-0.40 [-0.95, 0.16]). The findings indicated there was no improvement in anxiety or depression in the waiting list. | 7 |
| **DISCUSSION** | | |  |
| Summary of evidence | 24 | A total of nine records that included ten studies were included in our meta-analysis. we identified an effect of ICBT-i on comorbid anxiety and depression, despite the heterogeneity and limitations of the meta-analysis. | 7-9 |
| Limitations | 25 | **Limitations:** Firstly, the small number of studies and sample size limited the ability to draw more solid conclusions. Secondly, other methodological limitations existed in these studies which would resulted in heterogeneity. | 8-9 |
| Conclusions | 26 | **Conclusion：**In summary, we identified an effect of ICBT-i on comorbid anxiety and depression, despite the heterogeneity and limitations of the meta-analysis. Additional high quality studies with larger sample sizes are needed to provide further evidence. | 9 |
| **FUNDING** | | |  |
| Funding | 27 | This work was supported by the Science and Technology Achievement Transformation Fund of the Third Military Medical University (2014XHZ10). | 9 |

*From:*  Moher D, Liberati A, Tetzlaff J, Altman DG, The PRISMA Group (2009). Preferred Reporting Items for Systematic Reviews and Meta-Analyses: The PRISMA Statement. PLoS Med 6(6): e1000097. doi:10.1371/journal.pmed1000097

For more information, visit: **www.prisma-statement.org**.

Page 2 of 2
